# Admission Homocysteine as a Potential Predictor for Delayed Cerebral Ischemia After Aneurysmal Subarachnoid Hemorrhage

**DOI:** 10.3389/fsurg.2021.813607

**Published:** 2022-02-08

**Authors:** Tiesong Zhang, Luyuan Zhang, Kehui Nie, Jun Yang, Haiyan Lou, Jincheng Wang, Sicong Huang, Chenjie Gu, Min Yan, Renya Zhan, Jianwei Pan

**Affiliations:** ^1^Department of Neurosurgery, College of Medicine, The First Affiliated Hospital, Zhejiang University, Hangzhou, China; ^2^Neusoft Medical System, Shanghai, China; ^3^Department of Radiology, College of Medicine, The First Affiliated Hospital, Zhejiang University, Hangzhou, China; ^4^Department of Hepatobiliary and Pancreatic Surgery, College of Medicine, The First Affiliated Hospital, Zhejiang University, Hangzhou, China

**Keywords:** aneurysm, subarachnoid hemorrhage, delayed cerebral ischemia, homocysteine, biomarker

## Abstract

**Background:**

Delayed cerebral ischemia (DCI) is a primary cause of poor prognosis after aneurysmal subarachnoid hemorrhage (aSAH) and needs close medical attention in clinical practice. Homocysteine (Hcy) has been implicated in cerebrovascular diseases. This study aimed to investigate whether serum Hcy could help to predict the occurrence of DCI in aSAH patients, and compare its diagnostic value with traditional methods.

**Methods:**

We enrolled 241 aSAH patients in this study. Serum Hcy levels were collected from each patient. The baseline information was reviewed and analyzed. The binary logistic regression was used to explore the relation of serum Hcy levels with occurrence of DCI, and diagnostic performance of serum Hcy for predicting DCI was evaluated using a receiver operating characteristic (ROC) curve.

**Results:**

The admission serum Hcy levels were found significantly higher in aSAH patients with DCI than those without (*P* < 0.001). The serum Hcy levels were positively correlated with the World Federation of Neurosurgical Societies (WFNS) scores, modified Fisher scores as well as Hunt and Hess scores at admission. Multivariate analysis revealed that occurrence of DCI was associated with serum Hcy levels (Odds Ratio [OR] = 1.257; 95% Confidence Interval [CI], 1.133–1.396, *P* < 0.001), modified Fisher scores (OR = 1.871; 95%CI, 1.111–3.150, *P* = 0.018) and Hunt and Hess scores (OR = 2.581; 95%CI, 1.222–5.452, *P* = 0.013) after adjusting for the significant variables in univariate analysis. Meanwhile, serum Hcy levels achieved good performance for DCI prediction (area under the curve [AUC], 0.781; 95%CI, 0.723–0.831, *P* < 0.001).

**Conclusion:**

Serum homocysteine might have the potential to be a useful and cost-effective biomarker for predicting the occurrence of DCI in aSAH patients.

## Introduction

Aneurysmal subarachnoid hemorrhage (aSAH) not only causes instantaneously severe damage to patients but also results in a series of neurological dysfunctions in the later days (1). Delayed cerebral ischemia (DCI) as a primary cause of adverse outcomes after aSAH is characterized by the occurrence of focal neurological deficits that were not manifested on initial imaging. There are several possible pathophysiological mechanisms involved in DCI, including cerebral vasospasm, microvessel thrombosis, and microvascular dysfunction (2–5). DCI typically occurs at 3–14 days after the initial aSAH, and around 30% of patients with aSAH maybe suffer from DCI (6). Therefore, it is expected that more attention should be paid to DCI owing to its high incidence and unfavorable prognosis (7, 8).

Homocysteine (Hcy) is a sulfur-containing amino acid mainly produced during metabolism of S-adenosyl-L-methionine. Furthermore, the retardation of Hcy metabolism could result in an elevated level of serum Hcy, named hyperhomocysteinemia (HHcy). Prior research studies specifying the effect of HHcy have described an intimate connection with cerebrovascular diseases. It has been reported that HHcy is an independent risk factor of ischemic stroke, and others found that serum Hcy could predict the risk of acute cerebral infarction (9–11). Ji et al. (12) found that HHcy is predictive of severe neurological impairments or other poor functional outcomes in patients with acute ischemic stroke. However, the relationship between serum Hcy levels and the occurrence of DCI after aSAH remains unclear.

This study aimed to investigate whether serum Hcy levels obtained at admission in aSAH patients could help to predict the development of DCI, even after adjusting for several known predictive factors.

## Materials and Methods

### Study Population

We retrospectively enrolled 241 consecutive patients with aSAH admitted to the Department of Neurosurgery in the First Affiliated Hospital of Zhejiang University Medical College from January 2016 to December 2019. The inclusion criteria for aSAH patients were as follows: (1) patients admitted at the hospital within 48 h of SAH onset and diagnosed *via* computed tomography angiography (CTA) or digital subtraction angiography (DSA); and (2) the serum Hcy levels at admission of hospitalization were collected. The exclusion criteria were as follows: (1) radiographic evidence of DCI presented at admission; (2) a history of previous craniocerebral trauma or any hemorrhagic brain injury treated by craniotomy; and (3) other systemic diseases such as severe hepatic dysfunction, malnutrition, and malignant tumor. Our study was carried out following the Code of Ethics of the World Medical Association (Declaration of Helsinki). This single-center, retrospective, observational study was approved by the institutional ethics committee, and written informed consent was acquired from the patient or a surrogate.

### Clinical Management

The guidelines of the American Heart Association and the American Stroke Association were applied in this study for clinical management (13). Critical care management conformed to the guidelines of the Neurocritical Care Society (14). All patients received intravenous nimodipine (2.1 mg/h) and supplemental fluids since the day of admission to maintain euvolemia and prevent the potential vasospasm. Hypertensive hypervolemic therapy was applied to postoperative patients who suffered from severe angiographic vasospasm or symptomatic vasospasm by setting the target systolic blood pressure to 180–220 mmHg (15).

### Clinical Data, Aneurysmal Morphology, and Neurological Assessments

The information including age, gender, vascular risk factors (hypertension, diabetes, hyperlipemia, and currently smoking), mean arterial pressure (MAP), body mass index (BMI), laboratory examination such as international normalized ratio (INR) and serum Hcy levels, radiological measurements (modified Fisher scores, presence of intraventricular hemorrhage or not), aneurysmal characteristics (location, the largest diameter of the aneurysm, number of aneurysms), and treatment methods (coiling or clipping) were reviewed and obtained from the medical records of patients.

The neurological assessment at admission was evaluated using the World Federation of Neurosurgical Societies (WFNS) scores as well as the Hunt and Hess scores (16, 17). The neuroradiologic grade was radiologically classified by the volume and distribution of subarachnoid bleeding on the initial CT scans according to the modified Fisher scores (18). Delayed cerebral ischemia (DCI) was defined as: (1) the occurrence of focal neurological impairment (such as hemiparesis, aphasia, apraxia, hemianopia, or neglect), or a decrease in at least two points on the Glasgow Coma Scale that was not apparent immediately after aneurysm occlusion; (2) there is a new infarction on CT. Meanwhile, other potential causes of low-density area such as clipping treatment or absorption of a hematoma had been rigorously excluded (2, 19). The initial CT scans were independently analyzed and evaluated by a neurologist (Tiesong Zhang) and a neuroradiologist (Jincheng Wang) who were blind to the serum Hcy levels and baseline information of patients.

### Statistical Analysis

Receiver operating characteristic (ROC) curve analysis was done using MedCalc Statistical Software v19.3 (MedCalc Software Ltd, Ostend, Belgium; https://www.medcalc.org; 2020) and other statistical analyses were done using SPSS v22.0 (IBM, Armonk, NY, United States). Normally distributed continuous variables were expressed as mean ± standard deviation and *t*-test was used for the two-group comparison, while non-normally distributed continuous variables were expressed as median (interquartile range) and Mann–Whitney *U* test was used. The categorical variables were expressed as counts (percentage) and analyzed by either the χ^2^ test or Fisher's exact test. Spearman rank correlation test was used to assess bivariate correlations. The binary logistic regression analyses were used to analyze the relationship of the serum Hcy levels and DCI after controlling for known predictors, including age, gender, smoking, WFNS scores, Hunt and Hess scores, as well as modified Fisher scores (5, 7). Those variables with a *P-*value ≤ 0.05 in the univariate logistic regression and other important factors associated with the cerebrovascular disease were included in the multivariate analyses using stepwise backward selection. The *P*-value ≤ 0.05 was considered as statistically significant.

### Performance Assessments of Serum Hcy in Predicting DCI

The predictive values of variables for DCI were assessed by the area under the curve (AUC) in ROC curve analysis. Furthermore, the ROC curves among serum Hcy levels, modified Fisher scores, as well as Hunt and Hess scores were compared by using the *Z*-test. The *Z*-value ≥ 1.96 were considered statistically significant. In addition, a combined model consisting of Hcy, modified Fisher scores, and Hunt and Hess scores was established to better predict DCI. Meanwhile, the predictive value of the combined model was also evaluated *via* ROC curve analysis.

## Results

### Demographic and Clinical Features

**Figure 1:**
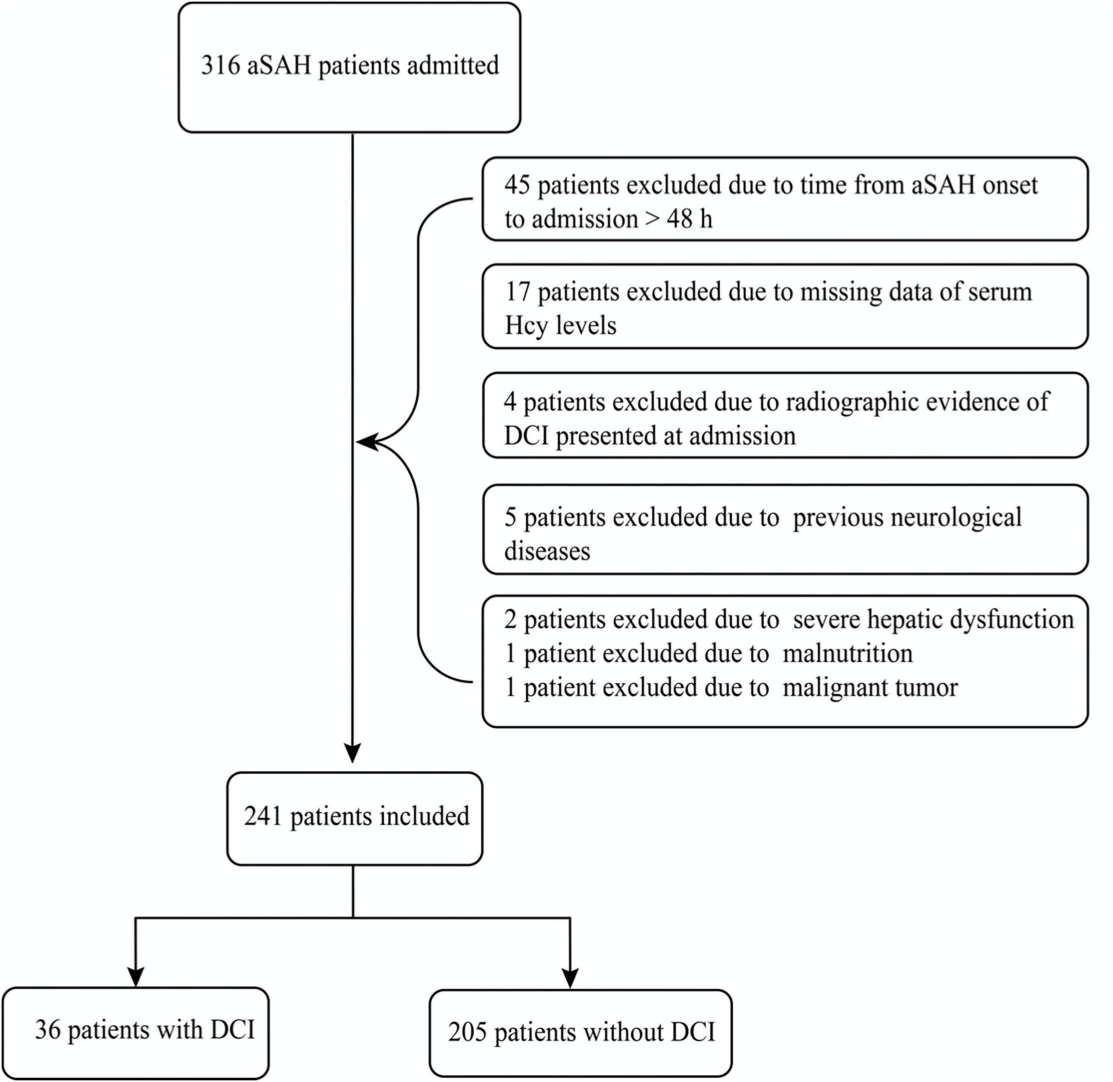
Flowchart of our study. A total of 241 patients with aneurysmal subarachnoid hemorrhage (aSAH) were finally included in this study, meanwhile, 36 patients suffered from delayed cerebral ischemia (DCI) days later.

A total of 316 aSAH patients were registered to this cohort during the study period. And then we excluded 75 patients because of some reasons listed in Figure 1. Ultimately, a total of 241 patients were included and analyzed. The mean age of the 241 aSAH patients was 56.8 ± 12.8 years (range, 20–85 years) and 53.5% were female. Comparison of demographics and baseline characteristics between the patients with DCI and without DCI are shown in Table 1. Patients with DCI were older (mean age, 60.9 ± 14.6 *vs*. 56.1 ± 12.2, *P* = 0.035); were more likely to smoke (36 cases, 41.7% *vs*. 205 cases, 24.9%, *P* = 0.037); had higher serum Hcy levels (median [interquartile range], 16.95 [12.29–20.2] *vs*. 10.5 [8.6–13.8], *P* < 0.001); exhibited higher admission WFNS scores (median [interquartile range], 3 [3–4] *vs*. 2 [1–3], *P* < 0.001), Hunt and Hess scores (median [interquartile range], 3 [3–4] *vs*. 2 [1–3]), and modified Fisher scores (median [interquartile range], 4 [3–4] *vs*. 2 [2–4], *P* = 0.002).

**Table 1:** The demographic and clinical data in patients with aneurysmal subarachnoid hemorrhage.

**Baseline characteristics**	**Overall** **(*n* = 241)**	**With DCI** **(*n* = 36)**	**Without DCI** **(*n* = 205)**	***P*** **-value**
**Demographics**				
Age (years)	56.8 ± 12.8	60.9 ± 14.6	56.1 ± 12.2	0.035
Gender (female)	129 (53.5%)	22 (61.1%)	107 (52.2%)	0.323
Hypertension	111 (46.0%)	15 (41.7%)	96 (46.8%)	0.567
Diabetes	14 (5.8%)	2 (5.6%)	12 (5.9%)	>0.99
Hyperlipemia	56 (23.2%)	6 (16.7%)	50 (24.4%)	0.312
Currently smoking	66 (27.4%)	15 (41.7%)	51 (24.9%)	0.037
**Clinical status on admission**				
MAP (mmHg)	100.0 (91.3–109.7)	100.2 (91.7–108)	99.7 (91.3–109.7)	0.945
BMI (kg/m^2^)	23.2 ± 3.0	23.1 ± 3.1	23.3 ± 3.0	0.905
INR	1.01(1.00–1.02)	1.02 (0.98–1.05)	1 (0.95–1.05)	0.553
Homocysteine (μmol/L)	11.1 (8.7–14.7)	16.95 (12.29–20.2)	10.5 (8.6–13.8)	<0.001
WFNS scores (continuous variable)	2 (1–3)	3 (3–4)	2 (1–3)	<0.001
WFNS scores (categorical variable)				<0.001
1	100 (41.5%)	4 (11.1%)	96 (46.8)	
2	61 (25.3%)	5 (13.9%)	56 (27.3%)	
3	21 (8.7%)	10 (27.8%)	11 (5.4%)	
4	38 (15.8%)	13 (36.1%)	25 (12.2%)	
5	21 (8.7%)	4 (11.1%)	17 (8.3%)	
Hunt and Hess scores (continuous variable)	2 (1–3)	3 (3–4)	2 (1–3)	<0.001
Hunt and Hess scores (categorical variable)				<0.001
1	86 (35.6%)	0 (0%)	86 (42.1%)	
2	58 (24.1%)	4 (11.1%)	54 (26.3%)	
3	58 (24.1%)	21 (58.3%)	37 (18.0%)	
4	32 (13.3%)	10 (27.8%)	22 (10.7%)	
5	7 (2.9%)	1 (2.8%)	6 (2.9%)	
**Radiological parameters**				
Modified Fisher scores (continuous variable)	3 (2–4)	4 (3–4)	3 (2–4)	0.002
Modified Fisher scores (categorical variable) variable)				0.002
1	50 (20.7%)	2 (5.6%)	48 (23.4%)	
2	29 (12.1%)	0 (0%)	29 (14.1)	
3	66 (27.4%)	14 (38.9%)	52 (25.4%)	
4	96 (39.8%)	20 (55.5%)	76 (37.1%)	
Intraventricular hemorrhage	125 (51.9%)	20 (55.6%)	105 (51.2%)	0.833
**Aneurysm characteristics**				
Aneurysms in anterior circulation	216 (89.6%)	32 (88.9%)	184 (89.8%)	0.774
Size of aneurysms (mm) ≤ 7	164 (68.0%)	24 (66.7%)	140 (68.3%)	0.679
Multiple aneurysms	50 (20.7%)	10 (27.8%)	40 (19.5%)	0.259
Aneurysm Treatment				0.114
Coil	118 (49.0%)	22 (61.1%)	96 (46.8%)	
Clip	123 (51.0%)	14 (38.9%)	109 (53.2%)	

*Values are shown as numbers (%) or median (IQR) or mean ± standard deviation. Abbreviations: DCI, delayed cerebral ischemia; MAP, mean arterial pressure; BMI, body mass index; INR, international normalized ratio; WFNS, World Federation of Neurosurgical Societies*.

### Serum Hcy Levels and Severity

**Figure 2:**
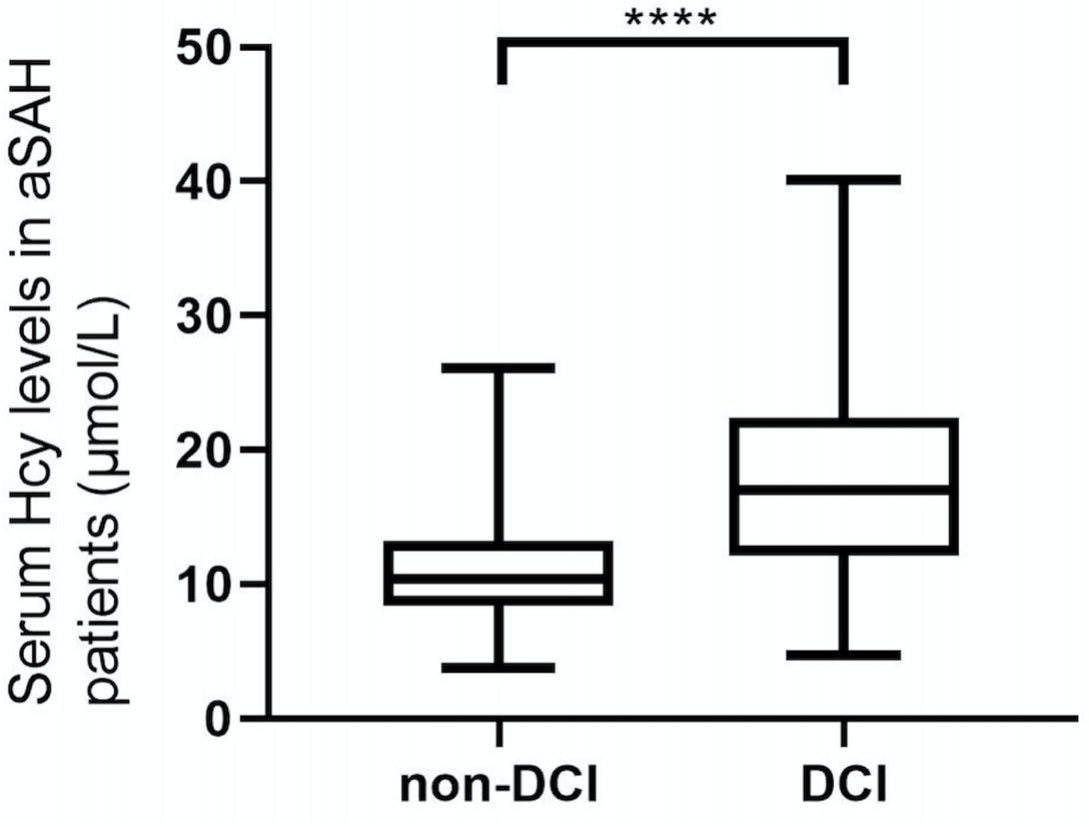
Difference in serum homocysteine (Hcy) levels between patients with DCI and patients without DCI. Patients with DCI had significantly higher serum Hcy levels than those without (*P* < 0.001). ^***^Statistical significance *P* < 0.001.

**Figure 3:**
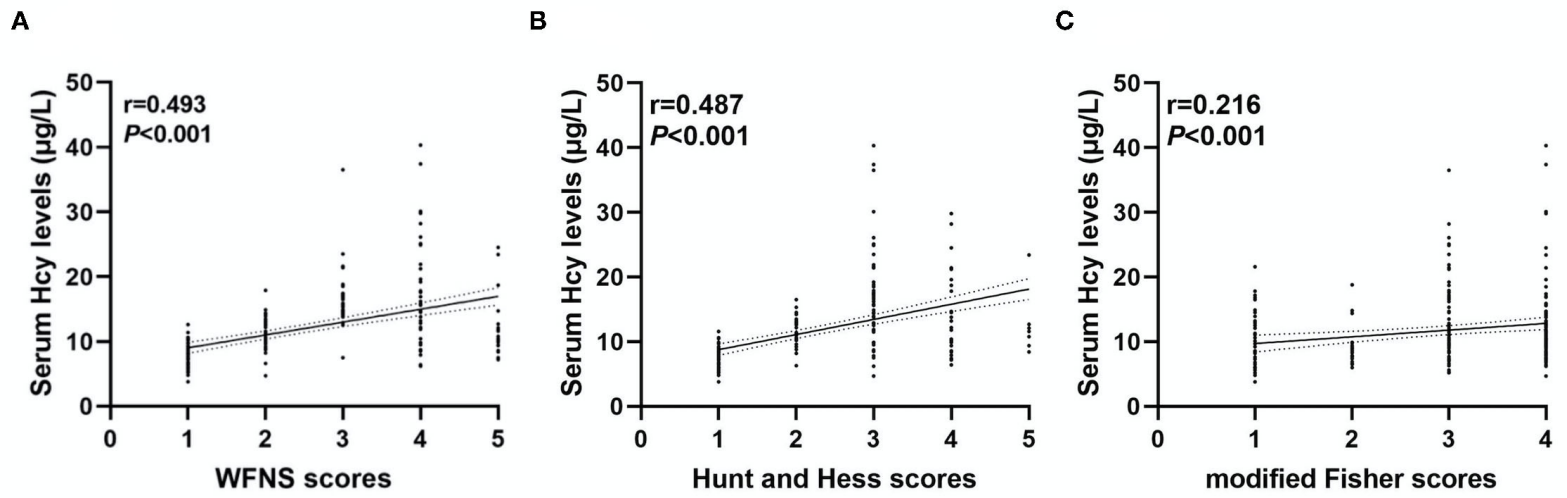
Correlation between serum homocysteine (Hcy) levels and clinical severity. Serum Hcy levels had a positive relationship with the World Federation of Neurosurgical Societies (WFNS) scores **(A)**, Hunt and Hess scores **(B)**, and modified Fisher scores **(C)**.

As shown in Figure 2, serum Hcy levels in patients with DCI were significantly higher. Table 1 showed that WFNS scores, as a categorical variable, had scores 1, 2, 3, 4, and 5 in 100, 61, 21, 38, and 21 aSAH patients, respectively. Similarly, the scores 1, 2, 3, 4, and 5 of Hunt and Hess scores were found in 86, 58, 58, 32, and 7 cases, respectively. Furthermore, the scores 1, 2, 3, and 4 of modified Fisher scores were revealed among 50, 29, 66, and 96 cases, respectively. Just as graphed in Figure 3, serum Hcy levels were positively correlated with WFNS scores, Hunt and Hess scores, as well as modified Fisher scores.

### Serum Hcy Levels and Incidence of DCI

A total of 36 patients (14.9%) developed DCI. Table 2 revealed that serum Hcy levels were still associated with DCI even if adjusting several important factors in different models. Furthermore, univariate analyses in Model 3 showed the risk factors associated with DCI were age, smoking, WFNS scores, modified Fisher scores, Hunt and Hess scores, as well as serum Hcy levels. And then, these above-mentioned variables and gender of patients were incorporated into a multivariate logistic regression model and subsequently, it showed that serum Hcy levels (OR = 1.257; 95%CI, 1.133–1.396, *P* < 0.001), modified Fisher scores (OR = 1.871; 95%CI, 1.111–3.150, *P* = 0.018), and Hunt and Hess scores (OR = 2.581; 95%CI, 1.222–5.452, *P* = 0.013) were the independent factors for the development of DCI (Table 3).

**Table 2:** Separate logistic regression models for serum homocysteine levels and delayed cerebral ischemia in patients with aneurysmal subarachnoid hemorrhage.

	**OR**	**95%Ci**	***P-*** **value**
Model 1	1.305	1.189–1.432	<0.001
Model 2	1.311	1.191–1.442	<0.001
Model 3	1.257	1.133–1.3965	<0.001

*Model 1, unadjusted. Model 2, adjusted for age and gender. Model 3, adjusted for age, gender, currently smoking, Hunt and Hess scores, World Federation of Neurosurgical Societies scores, and modified fisher scores*.

**Table 3:** Univariate and multivariate logistic regression analysis of predictors in Model 3 for delayed cerebral ischemia after aneurysmal subarachnoid hemorrhage.

	**Univariate analysis**		**Multivariate analysis**	
	**OR 95%CI**	***P*** **-value**	**OR 95%CI**	***P*** **-value**
Age (years)	1.031 (1.000–1.060)	0.037	—	—
Gender (female)	1.439 (0.698–2.969)	0.324	—	—
Currently smoking	2.157 (1.040–4.500)	0.040	—	—
Hunt and Hess scores	2.379 (1.687–3.355)	<0.001	2.581 (1.222–5.452)	0.013
WFNS scores	1.772 (1.370–2.292)	<0.001	—	—
Modified Fisher scores	1.905 (1.268–2.862)	0.002	1.871 (1.111–3.150)	0.018
Homocysteine	1.311 (1.199–1.433)	<0.001	1.257 (1.133–1.396)	<0.001

*WFNS, World Federation of Neurosurgical Societies*.

### Prediction Models for Predicting DCI

**Figure 4:**
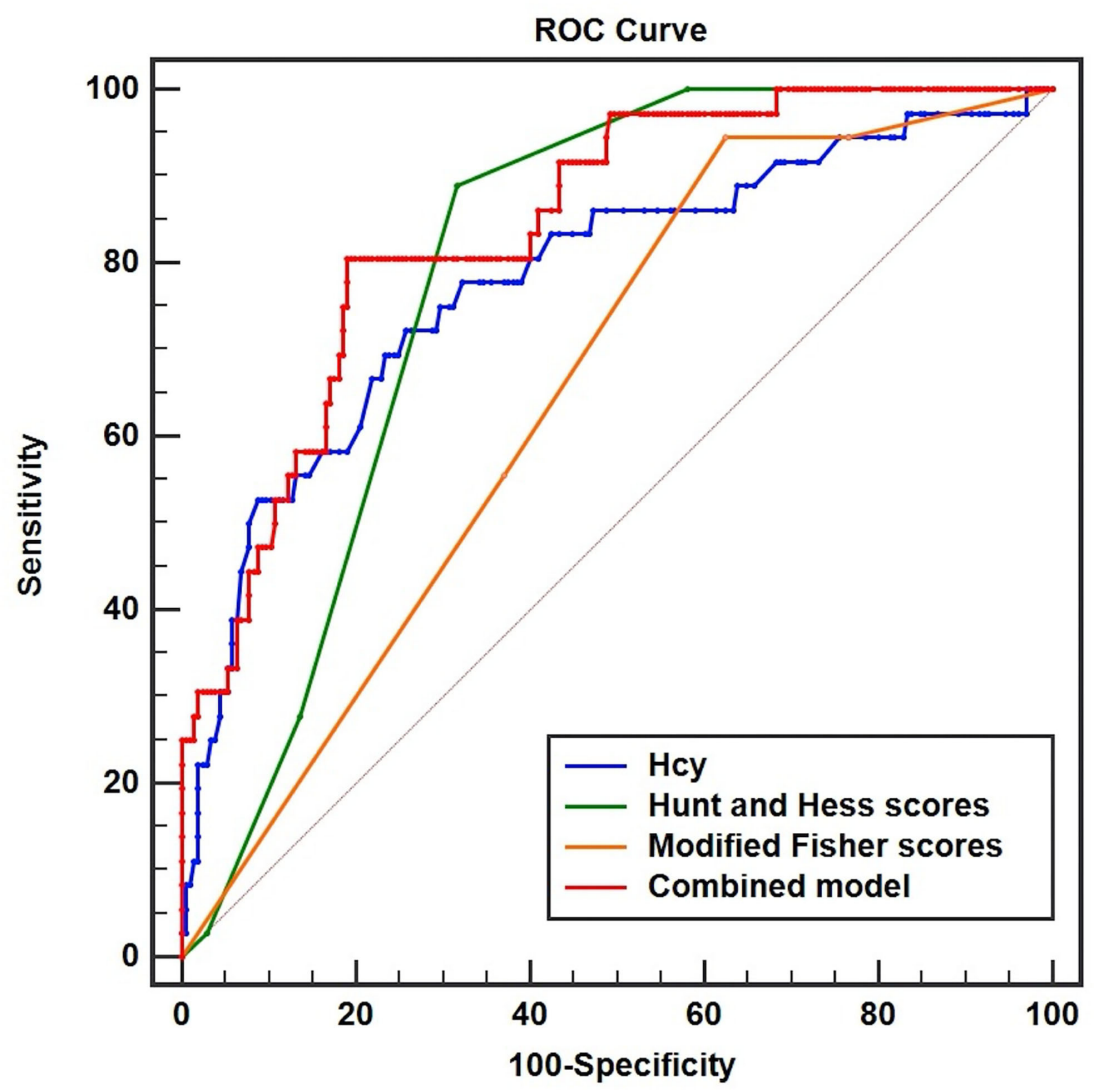
Receiver operator characteristic curve analysis comparing serum homocysteine (Hcy) levels, Hunt and Hess scores, modified Fisher scores, and combined model (Hcy/Hunt and Hess scores/modified Fisher scores) on predicting DCI in patients with aSAH. The area under the curves of serum Hcy levels, Hunt and Hess scores, modified Fisher scores, and combined model were 0.781 (95%CI, 0.723–0.831, *P* < 0.001), 0.790 (95%CI, 0.733–0.840, *P* < 0.001), 0.654 (95%CI, 0.591–0.714, *P* < 0.001), and 0.842 (95%CI, 0.789–0.885, *P* < 0.001), respectively.

In Figure 4, the AUC of serum Hcy levels for the prediction of the development of DCI was 0.781 (95%CI, 0.723–0.831, *P* < 0.001; Youden index = 0.4637). The serum Hcy levels of 13.5 μmol/L were regarded as the best cutoff value, which yielded sensitivity and specificity values of 72.22 and 74.15%, respectively. In addition, our study revealed that modified Fisher scores and Hunt and Hess scores were the other independent factors, whose AUC were 0.654 (95%CI, 0.591–0.714, *P* < 0.001, Youden index = 0.3201) and 0.790 (95%CI, 0.733–0.840, *P* < 0.001, Youden index = 0.5718), respectively. We concluded that the predictive performances of serum Hcy levels were equivalent to Hunt and Hess scores (*Z* = 0.205, *P* = 0.8373), but better than modified Fisher scores (*Z* = 2.146, *P* < 0.05). Interestingly, the combined model (Hcy/modified Fisher scores/Hunt and Hess scores) showed better predictive performances (AUC, 0.842; 95%CI, 0.789–0.885, *P* < 0.001, Youden index = 0.6153) with sensitivity and specificity values of 80.56 and 80.98%, respectively.

## Discussion

Subarachnoid hemorrhage can lead to a serial of physiologic disorders including increased intracranial pressure, decreased cerebral blood flow, particularly the cytotoxic components released from blood directly to the central nervous system during SAH (20). Lots of theories of mechanisms contribute to the development of DCI (21–23). Certain biochemicals such as procalcitonin and cerebrospinal fluid glutamate are, to some degree, correlative with the progression of poor prognoses in aSAH patients (24, 25). Furthermore, Serum Hcy levels have been found to play a critical role in facilitating the occurrence and progression of cerebral infarction, while a high level of serum Hcy is also associated with the risk of recurrence and adverse prognosis of cerebrovascular diseases (26–28). However, there is currently little evidence on the relationship between elevated serum Hcy levels and DCI after aSAH. Our study concentrates on exploring a more readily available biomarker, acquired from routine blood tests, which hopefully could be used as an indicator for clinicians in preventing the development of DCI and lowering the total risk of further brain injury after aSAH.

Elevated serum Hcy levels were attributed to Hcy metabolism disorders, including hereditary factor (mass synthesis of key enzyme), taking certain medicines (i.e., immunosuppressant and antiepileptic drugs), unhealthy living habits, as well as nutritional deficiencies in Vitamin B6, B12, and folic acid (29). As a biomarker of certain cardiovascular and cerebrovascular diseases such as occlusive arterial diseases, myocardial infarction, and cerebral infarction (26, 30, 31), serum Hcy indeed has an intimate connection with vascular diseases, making it a proper candidate to assess the clinical outcomes of aSAH (22, 28).

Our study demonstrates that Hcy may be involved in the occurrence and development of DCI after aSAH. Hendrix et al. (32) found that cystathionine beta-synthase polymorphisms could influence the levels of homocysteine and hydrogen sulfide that affect the prognosis in aSAH patients. However, given the limited evidence on pathophysiology, the deep-rooted mechanisms and principles of how Hcy is positively associated with DCI remains unclear. In addition, Hcy is considered to have influences on ischemic diseases by the following mechanisms; the elevated serum Hcy levels can promote cerebral vasospasm by increasing the apoptosis of endothelial progenitor cells (33, 34). Moreover, Hcy could significantly enhance the activity of cell surface tissue factor to intensify microvessel thrombosis (35). Besides, reactive oxygen species production stimulated by Hcy could further enhance oxidative stress and promote endothelial dysfunction (36). Furthermore, therapies for lowering the serum Hcy levels have been applied to those people with elevated Hcy levels which in turn help in minimizing the high risk of ischemic diseases (37). These well-known mechanisms and corresponding therapies may illustrate and suggest a potential treatment strategy tailored to the aSAH patients with elevated serum Hcy levels.

We not only found the positive correlation of serum Hcy levels with severity, but also revealed their close association with the development of DCI in aSAH patients after adjusting for age, gender, currently smoking, Hunt and Hess scores, WFNS scores, and modified Fisher scores. For predicting DCI, the AUC of serum Hcy levels was 0.781 (95%CI, 0.723–0.831, *P* < 0.001), and the optimal cutoff value for serum Hcy levels was 13.5 μmol/L based on the Youden index. Although total volumes and distribution of intracranial bleeding play a crucial role in the development of DCI, there is a strong association between the clinical status at admission and DCI, and several experimental research studies have examined several prediction models to improve the predictive accuracy of DCI by incorporating the Hunt and Hess scores and modified Fisher scores (38, 39). We found the effective abilities of three critical parameters (Hcy/ modified Fisher scores/ Hunt and Hess scores) to predict DCI as shown in our study, demonstrating that these above-mentioned indicators were independent risk factors of DCI. It was gratifying that the combined model had a significantly better predictive performance. However, compared with other prior reports, the real world showed by our data suggested that the modified Fisher scores in this study had a less predictive accuracy of DCI (39, 40). Although several studies revealed that patients with DCI possessed higher modified Fisher scores (40, 41), we found that the sensitivity of modified Fisher scores for predicting DCI was high and specificity was low (sensitivity, 94.44%; specificity, 37.56%). Moreover, Christopher Melinosky et al. (42) believed that there was an existing moderate agreement in grading the modified Fisher scores among raters, and emphasized a need to further evaluate the reliability and effectiveness of online training tools to standardize scoring criteria. In summary, our study revealed that serum Hcy levels at admission were significantly elevated in the aSAH patients who had DCI presented later. This observation indicated that serum Hcy might be used as a potential biomarker for predicting DCI in patients with aSAH.

Several limitations of this study need to be considered. First, there might have been an inherent risk of selection bias for the reason that we selected patients in this single-center, retrospective, observational cohort. Second, it had been shown that serum Hcy levels were significantly higher in aSAH patients with DCI than those without. However, because of the absence of any evidence, how serum Hcy levels varied before and after the onset of aSAH remained uncertain and should be further investigated. Last but not the least, our study revealed that serum Hcy levels have a positive correlation with severity at admission. However, we could not conclude whether there was a specific causal relationship between the serum Hcy levels and the degree of clinical nerve function damage. Therefore, future research studies should include exploration and identification of temporal changes of serum Hcy levels throughout the whole hospital course to explicate their dynamic effects on aSAH severity and prognosis.

## Conclusions

Admission serum Hcy may be used as an easily measured biomarker to predict the development of DCI in aSAH patients even after adjusting for the known predictors, which reminds us that the serum Hcy may be an underlying therapeutic target. We expect that early and continuing interventions to lower the serum Hcy levels might further reduce the incidence of DCI after aSAH and improve clinical prognosis.

## Data Availability Statement

The original contributions presented in the study are included in the article/Supplementary Material, further inquiries can be directed to the corresponding author/s.

## Ethics Statement

Written informed consent was obtained from the individual(s) for the publication of any potentially identifiable images or data included in this article.

## Author Contributions

JP, JY, and HL conceived the research contents. JW, SH, and CG collected the information and did data analysis. TZ, LZ, and KN completed the drafting of the manuscript. Finally, the manuscript was critically revised by RZ and MY. All authors contributed to this article and approved the final submitted version.

## Funding

This research was supported by the Key Research & Development (R&D) Plan of Zhejiang Province (No. 2019C03034).

## Conflict of Interest

Authors KN and JY were employed by Neusoft Medical System. The remaining authors declare that the research was conducted in the absence of any commercial or financial relationships that could be construed as a potential conflict of interest.

## Publisher's Note

All claims expressed in this article are solely those of the authors and do not necessarily represent those of their affiliated organizations, or those of the publisher, the editors and the reviewers. Any product that may be evaluated in this article, or claim that may be made by its manufacturer, is not guaranteed or endorsed by the publisher.
